# Continuous cement leakage along the posterior longitudinal ligament of the intraspinal epidural during a percutaneous vesselplasty: A case report and literature review

**DOI:** 10.3389/fsurg.2022.1087591

**Published:** 2023-01-09

**Authors:** Ning An, Sijia Guo, Jisheng Lin, Haoxiang Zhuang, Hai Meng, Nan Su, Qi Fei

**Affiliations:** Department of Orthopedics, Beijing Friendship Hospital, Capital Medical University, Beijing, China

**Keywords:** cement leakage, complication, percutaneous kyphoplasty (PKP), epidural, classification standard

## Abstract

**Objective:**

This study aims to report one case of intraspinal epidural cement leakage caused by a novel percutaneous vesselplasty.

**Methods:**

A clinical case report from the Orthopedic center of our hospital and a literature review. A 63-year-old woman with an L_2_ osteoporotic compression fracture underwent novel kyphoplasty, percutaneous vesselplasty. This rare complication was evaluated through a literature search, and its special types are classified in more detail.

**Results:**

The patient was hospitalized with low back pain two weeks after a fall. After auxiliary examination, a new type of percutaneous vesselplasty was performed. After the intraoperative injection of bone cement, bone cement leakage extended along the posterior longitudinal ligament and epidural space. There were no special compression symptoms of the spinal cord, and the prognosis of conservative treatment was good.

**Conclusion:**

Although percutaneous vesselplasty is relatively safe and frequent, intraspinal leakage may occur, so sufficient preoperative evaluation, intraoperative continuous fluoroscopic monitoring, and timely evaluation of postoperative images are extremely necessary.

## Introduction

Percutaneous vertebroplasty (PVP) and percutaneous kyphoplasty (PKP) are effective methods for osteoporotic vertebral compression fractures. However, bone cement leakage is a common complication. Some types of bone cement leakage will not cause serious consequences and are mostly asymptomatic, but some will cause serious consequences, even irreversible injury (1). Herein, we report a special case in which the bone cement infiltrated the epidural space in the spinal canal during percutaneous vesselplasty and spread along the posterior longitudinal ligament, emphasizing the potential risks of this type of percutaneous vesselplasty.

## Case report

The patient was a 63-year-old female who complained of low back pain after a fall for two weeks. The admission examination showed no obvious deformity of the lumbar vertebrae, positive tenderness and percussion pain of the lumbar spinous process, obvious limitation of lumbar movement, no obvious abnormality of sensory muscle strength of the lower extremities, and no pathological signs. The bone scan showed abnormal imaging agent concentration and compressibility in L2 vertebral body. The main diagnosis was osteoporotic vertebral compression fracture (L2), and VAS pain score was 7, so a percutaneous vesselplasty was planned (2, 3).

**Figure 1:**
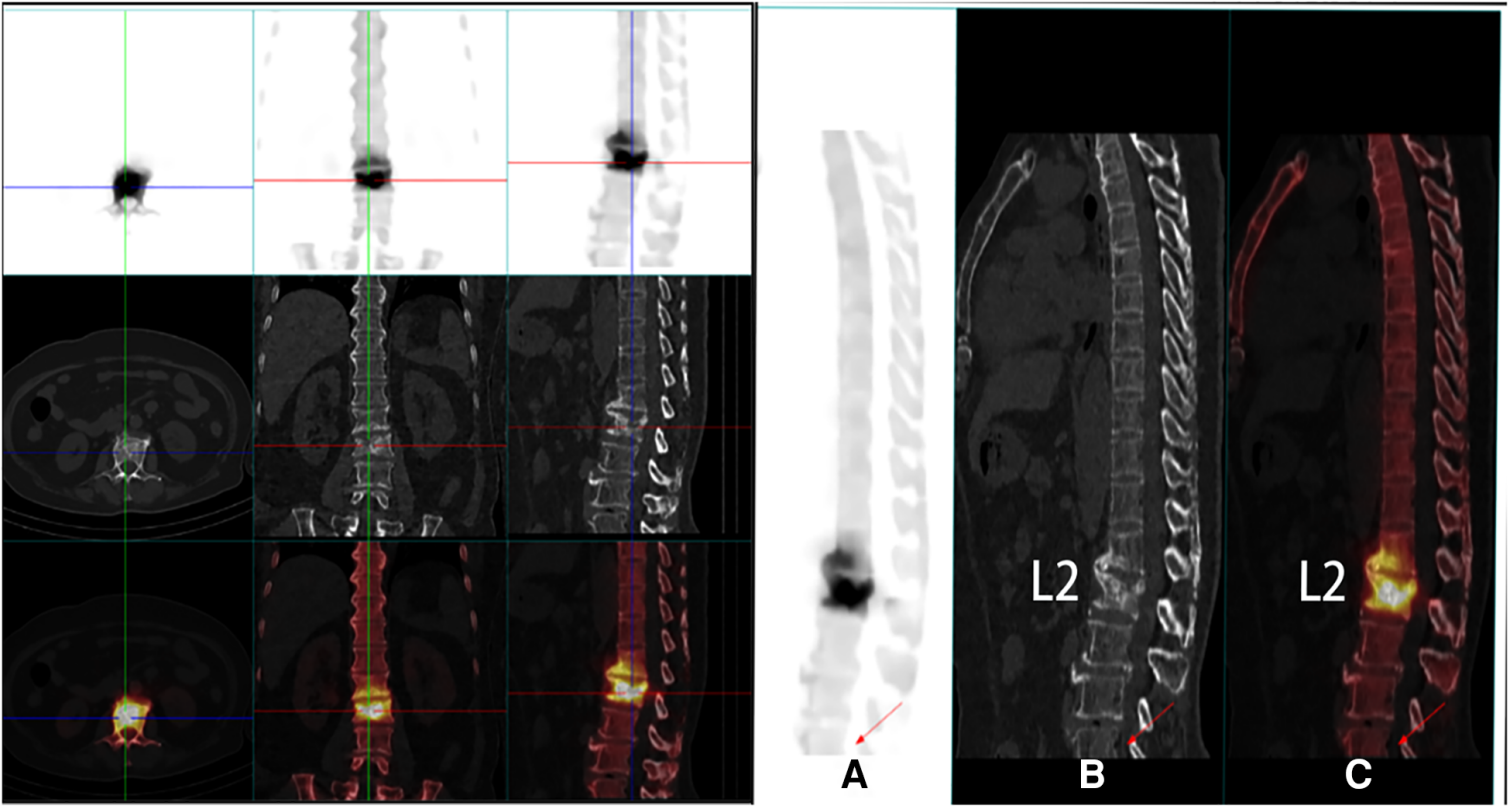
PET-CT images of the patient's preoperative coronal and sagittal vertebral fractures (**A–C**).

**Figure 2:**
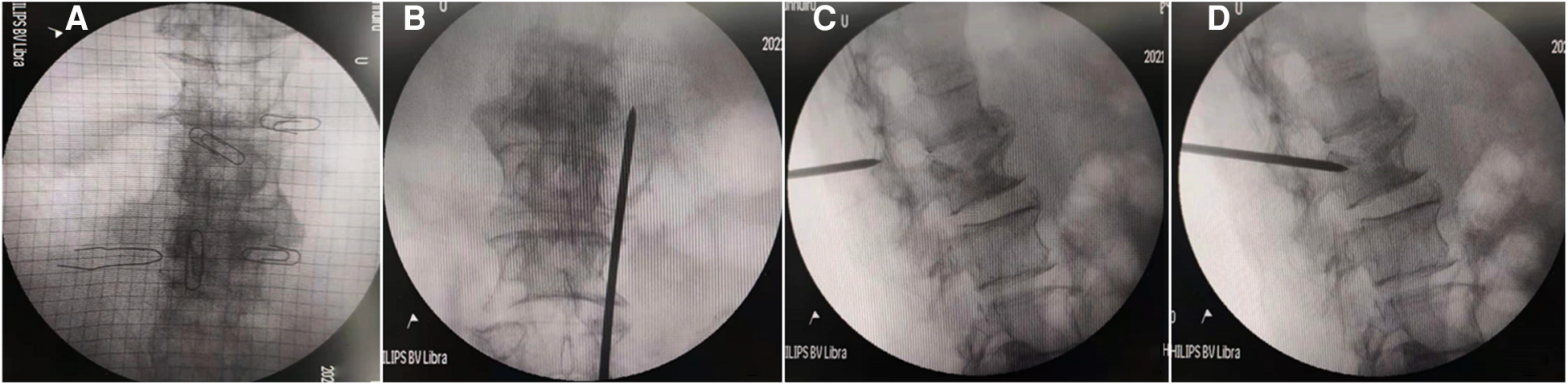
Intraoperative continuous fluoroscopy before the bone cement leakage (**A–C** front and Side view, **D** puncture needle entering the vertebral body through the pedicle).

**Figure 3:**
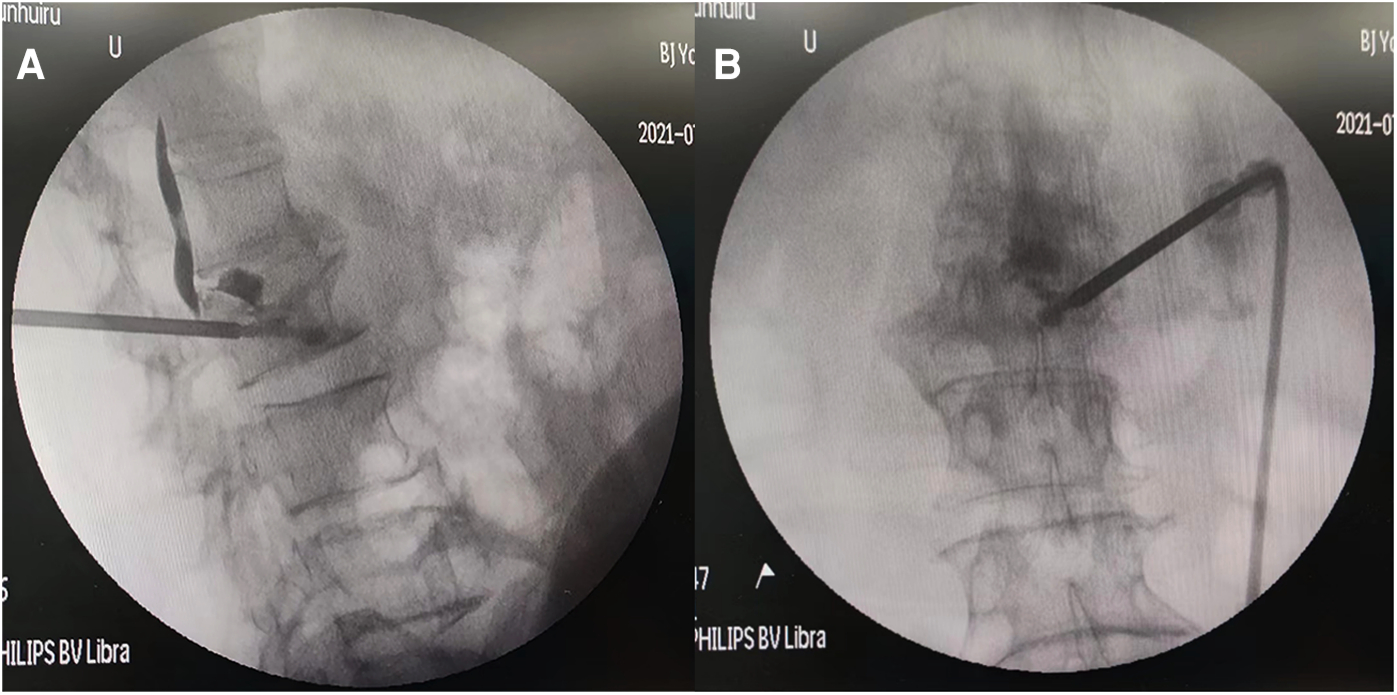
Intraoperative fluoroscopy after bone cement leakage (**A,B** front and Side view).

We used the related instruments of the vertebroplasty system (VCF-XTM Bone Filler Delivery System), including a double-layer bone filling bag BVFX-D20, controllable bone cement injection device, extension tube TmurCCD2murT301, bone drilling needle and working casing TmurN201T, as well as a precision bit T-D401 (4). The patient was lying prone, and the involved vertebral segments were located by Kirschner wire and C-arm x-ray machine fluoroscopy. The entry point was marked, disinfected, and local anesthesia applied. The bone scan and tomography showed abnormal concentration and compressibility of imaging agents in L_2_ vertebrae (Figure 1). At the beginning of the operation, the puncture needle was inserted through the right pedicle until it reached the midline of the vertebral body in the positive position (Figure 2) (5). For more satisfactory dispersion, we chose to break the bone filling bag. At this time, the patient developed transient low back pain, which was relieved after a few minutes, and fluoroscopy showed that the bone cement extended along the posterior longitudinal ligament space and epidural space (Figure 3). The patient's vital signs were good, with no obvious abnormality in the lower limb movement, and the operation was stopped. After the operation, the patient returned to the ward and was given symptomatic treatment and monitored.

**Figure 4:**
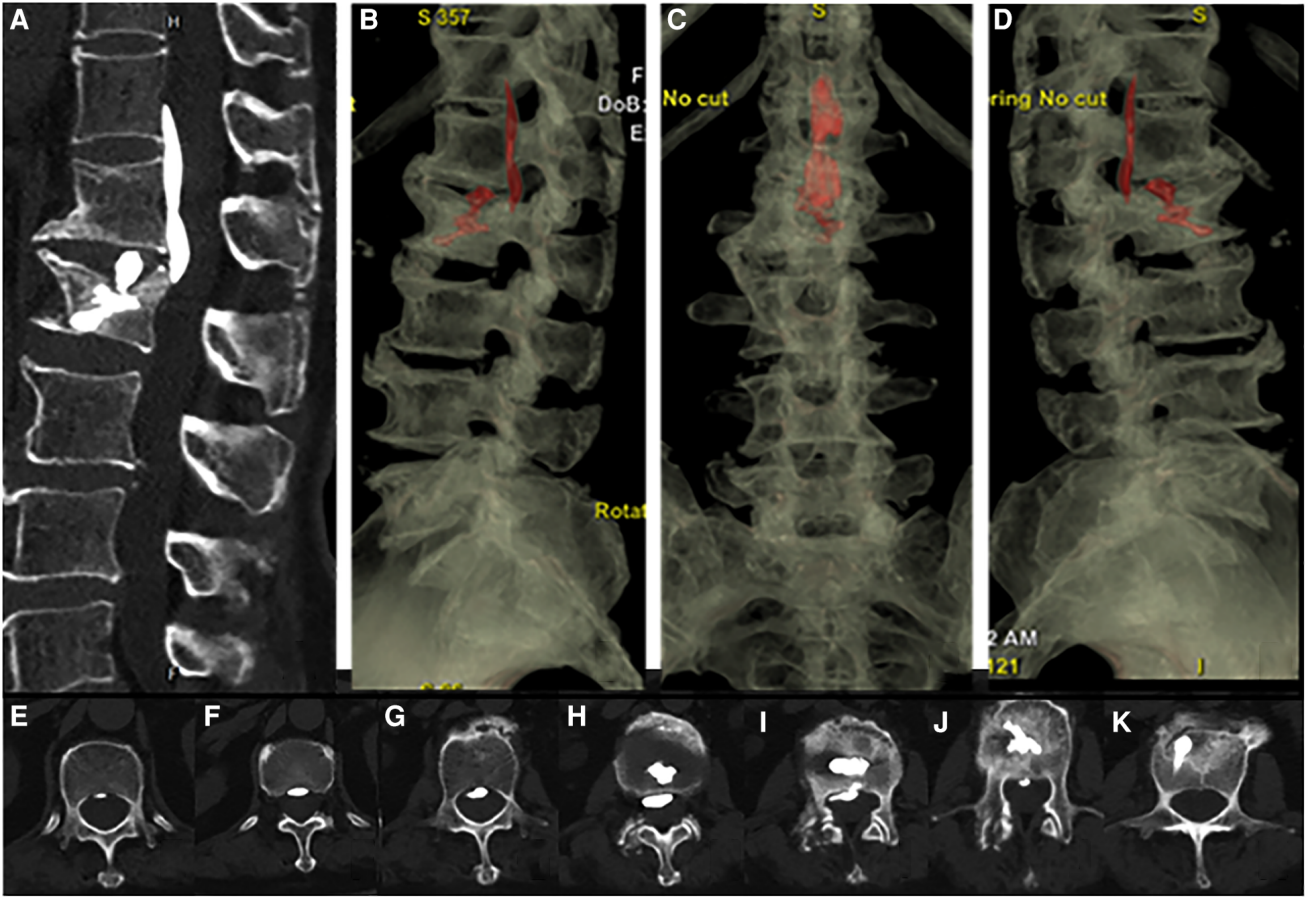
Postoperative CT scan 3D reconstruction showed bone cement infiltrating the epidural space of the spinal canal and distributed along the posterior longitudinal ligament from T12 to L2 (**A–D**) and the filling of bone cement in the posterior spinal canal of the L2 vertebral body(**E–K**).

Her low back pain was significantly better than before the operation, and her VAS pain score decreased from 7 to 2. There was no obvious movement and sensory disturbance, and urination and defecation were normal. A routine CT scan after operation showed that L2 vertebral body was filled with bone cement, and there was a small amount of bone cement leakage in L1–2 intervertebral disc, which leaked into the epidural space of the spinal canal and distributed along the posterior longitudinal ligament from T12 to L2. There was no compression of the spinal cord, as shown in Figure 4.

After routine treatment, all laboratory indexes were in the normal range at discharge. Routine follow-up was performed three and six months after the operation, and the patient recovered well without discomfort. Because this type of cement leakage is rare, we will continue to follow up the patient, and we will detect other discomfort in time if there is any subsequent discomfort.

## Discussion

Percutaneous kyphoplasty (PKP), as minimally invasive spine surgery, has a long history of development and mature technology (6, 7). It is widely used for its advantages, such as less trauma and good postoperative effect (8). Notably, percutaneous vesselplasty is a further surgical approach based on PKP (9, 10). The progress of technical without affecting the diffusion of bone cement greatly reduce the possibility of cement leakage into the spinal canal through the fracture line or the posterior edge of discontinuous vertebral body and reduce the most serious neurological complications (11).

It has been reported that bone cement leakage is one of the most common complications in PKP surgery, with an incidence of about 15%. The different leakage sites and leakage paths can be classified into several types. Among them, the consequences of intraspinal leakage are the most serious and therefore have received more attention (12). Cement protruding into the spinal canal may cause compression of nerves, numbness and pain in the lower limbs, and even paralysis.

There are several common types of bone cement leakage. The first is Yeom classification: *via* a basivertebral vein (type B), *via* a segmental vein (type S), and through a cortical defect (type C), in which type C is refined into two subtypes with leakage reaching the intervertebral disc (type D) and leakage not reaching the intervertebral disc (type C) (13). The second classification is the Wang classification, including five types: type A, through a cortical defect into the paraspinal soft tissues; type B, through the basivertebral foramen; type C, *via* the needle channel; type D, through a cortical defect into the disc space; and type E, *via* the paravertebral vein (14). According to the existing classification criteria, our case should be included in the compound type D that enters the intervertebral disc space through cortical damage. However, in the above traditional classification, there is no specific classification of intraspinal leakage (15, 16); thus, because of the particularity of this case report, we recommend a more specific classification of bone cement leakage into the bony spinal canal. Division, according to its anatomy, and the results of compression of the dural sac will be of guiding significance to whether and when we should take measures.

The posterior longitudinal ligament is composed of two layers, and the fibers from the deep layer are closely combined with the fibers of the annulus fibrosus of the intervertebral disc (17). There is a potential fascial fissure of considerable size on the dorsal side (18). The posterior part of the superficial layer of the posterior longitudinal ligament is connected to the dura mater, which effectively prevents the leakage of bone cement into the dural space (19). Therefore, according to its leakage path, it is reasonable to classify it as the intraspinal epidural space, and because of its complex leakage path, it is more appropriate to classify it as a mixed leakage.

We believe that it is extremely necessary to determine whether the leakage is located inside or outside the dura and the extent of compression. Only when lower limb symptoms occur, do we recommend acute decompression. Open decompression through a posterior approach or spinal endoscopy should be performed to relieve compression (20). Consequently, given the more uniform shape of the leak in our case, which is located in the epidural intraspinal canal, we believe that conservative treatment can be successful, such as rest immobilization and anti-inflammatory analgesia.

X-rays are routinely used to evaluate the intraoperative process and distribution of bone cement, which may lead to a missed diagnosis. Early and faster intervention is recommended for patients with bone cement leakage; therefore, performing intraoperative CT scanning and neurophysiological examinations when necessary for special types of bone cement leakage rather than simply classifying patients by symptoms (21).

By exploring the reasons for the leakage of bone cement in this case, combined with the current clinical experience, the preventive measures for the type of leakage may include the following: an improved preoperative examination including various necessary imaging and physical examinations to identify the anatomy of the fractured vertebral body, the degeneration of the upper and lower intervertebral discs, and the integrity of the vertebral body (22, 23). Due to the different injection methods of bone cement in the balloon, a more careful operation is required, the amount of bone cement bolus should also be appropriate, and continuous fluoroscopic monitoring is recommended (24–26). We suggest that we should be more cautious in the treatment of special types of fractures or for which the type of injury cannot be fully determined by preoperative examination.

In summary, we suggest that the classification of intraspinal bone cement leakage should be more specific and clinical, and adequate preparations should be made before surgery to standardize intraoperative operations to prevent such complications as much as possible. Once such complications occur, the detailed types and ways of leakage can be mastered to predict the results more accurately. Patients can benefit more by taking appropriate measures according to the situation.

## Data Availability

The raw data supporting the conclusions of this article will be made available by the authors, without undue reservation.
